# Supplementary material for: Fam49b dampens TCR signal strength to regulate survival of positively selected thymocytes and peripheral T cells
Source: eLife. 2024 Aug 19;13:e76940. doi: 10.7554/eLife.76940 (PMC11333044; doi:10.7554/eLife.76940)
Supplement: Figure 1—source data 2. [file elife-76940-fig1-data2.zip › 1C,1D_Immunoblot for Fam49a with labeling.pptx]

## Slide 1
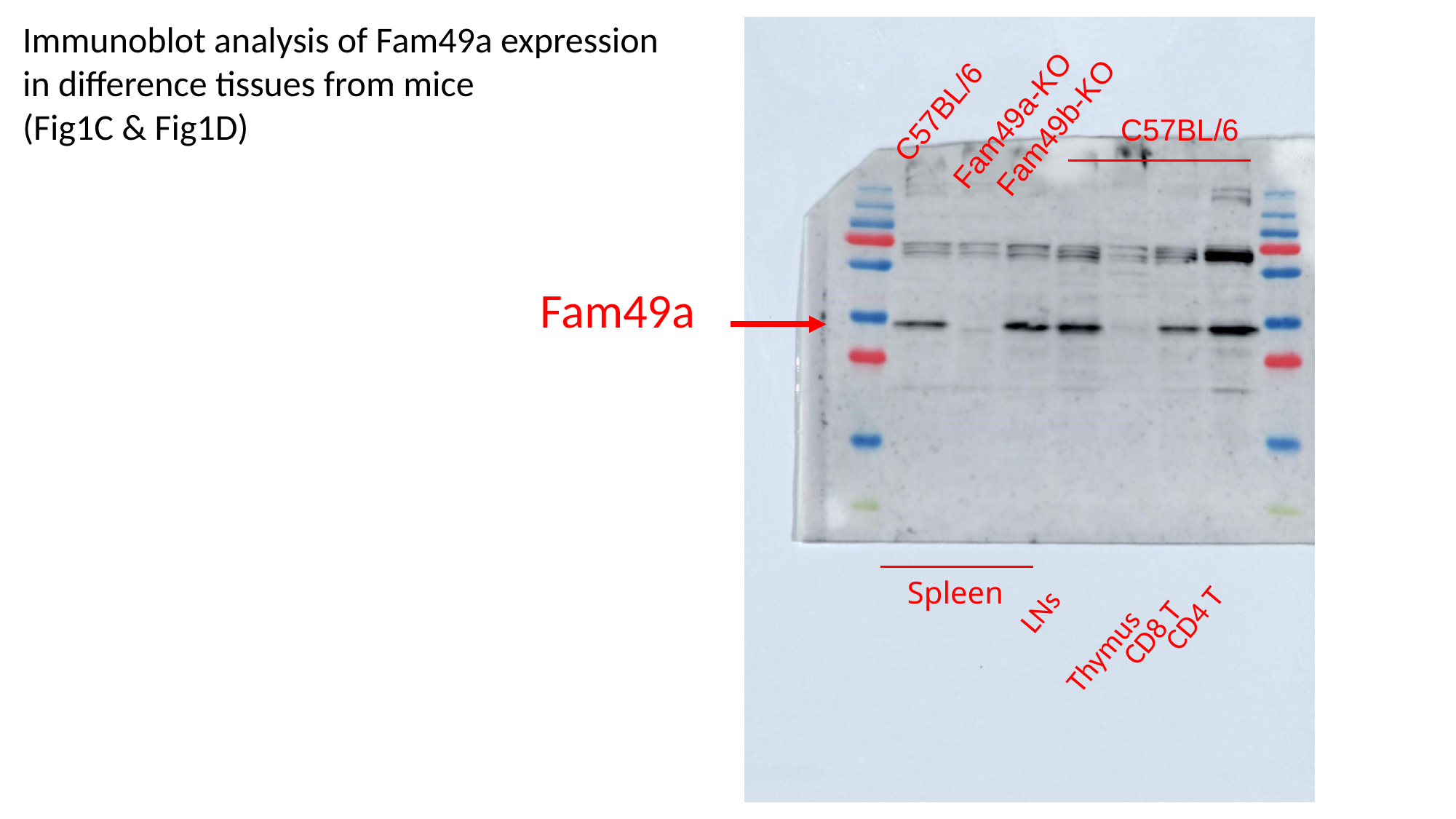

Immunoblot analysis of Fam49a expression
in difference tissues from mice
(Fig1C & Fig1D)
C57BL/6
Fam49a-KO
Fam49b-KO
C57BL/6
Fam49a
LNs
Spleen
CD4 T
CD8 T
Thymus
