# Supplementary figures and images for: Fam49b dampens TCR signal strength to regulate survival of positively selected thymocytes and peripheral T cells
Source: eLife. 2024 Aug 19;13:e76940. doi: 10.7554/eLife.76940 (PMC11333044; doi:10.7554/eLife.76940)

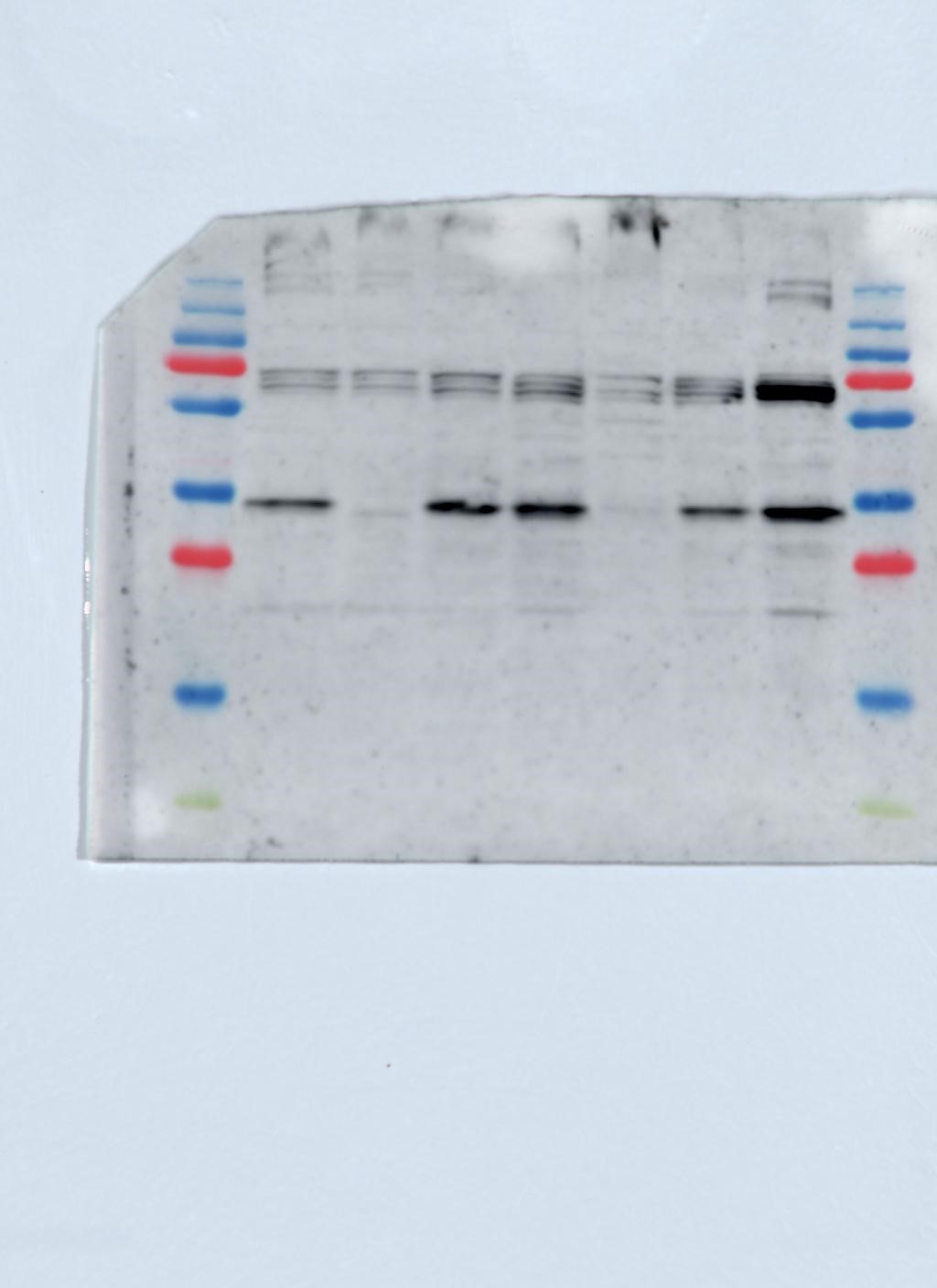

Supplement: Figure 1—source data 2. [file elife-76940-fig1-data2.zip › 1C, 1D_Immonoblot for Fam49a.jpg]

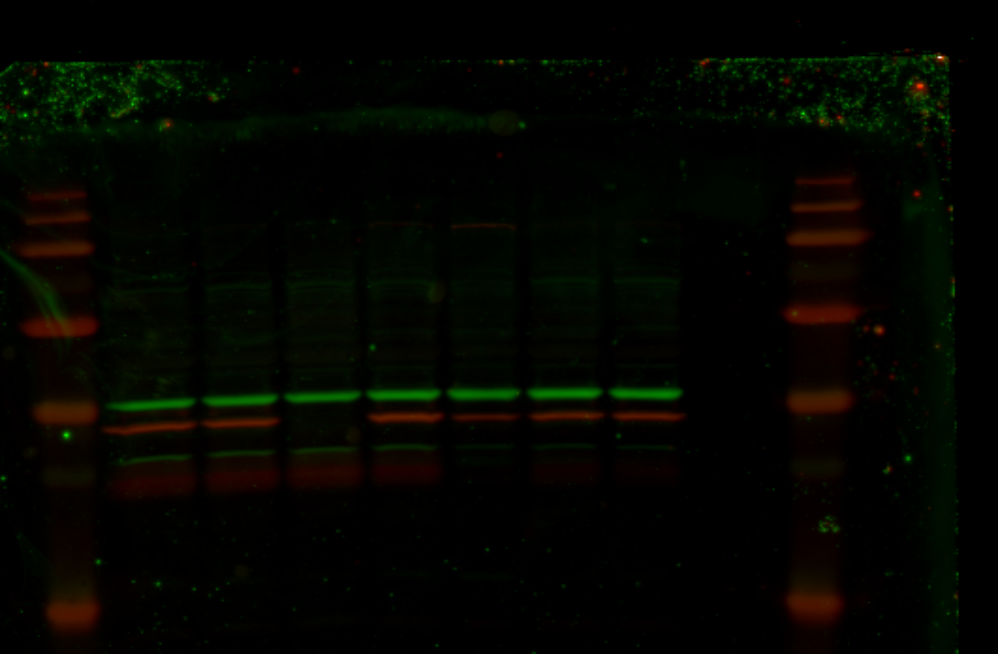

Supplement: Figure 1—source data 2. [file elife-76940-fig1-data2.zip › 1C, 1D_Immunoblot for Fam49b.png]

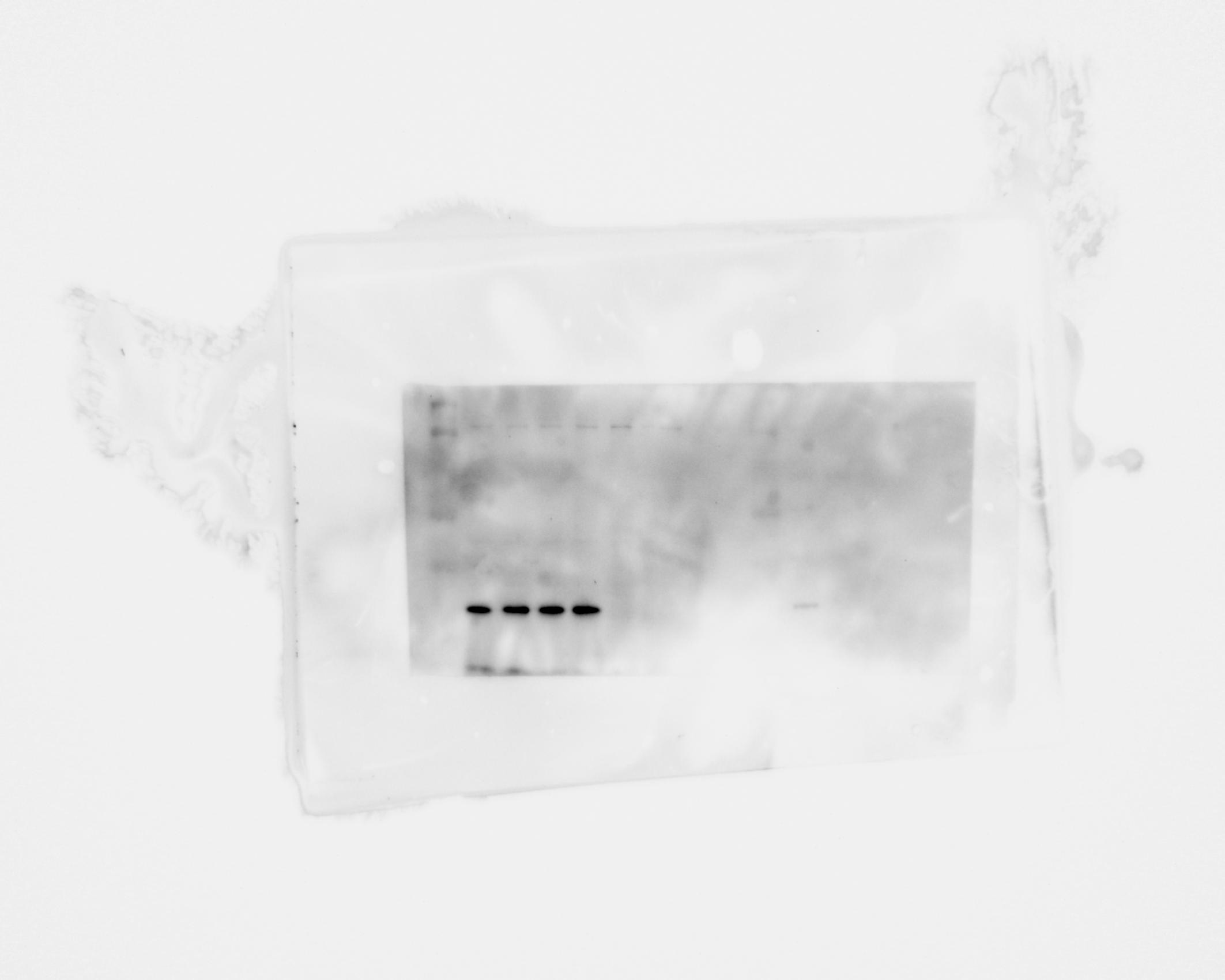

Supplement: Figure 4—source data 2. [file elife-76940-fig4-data2.zip › Figure 4 - source data 2/FAM49B.tif]

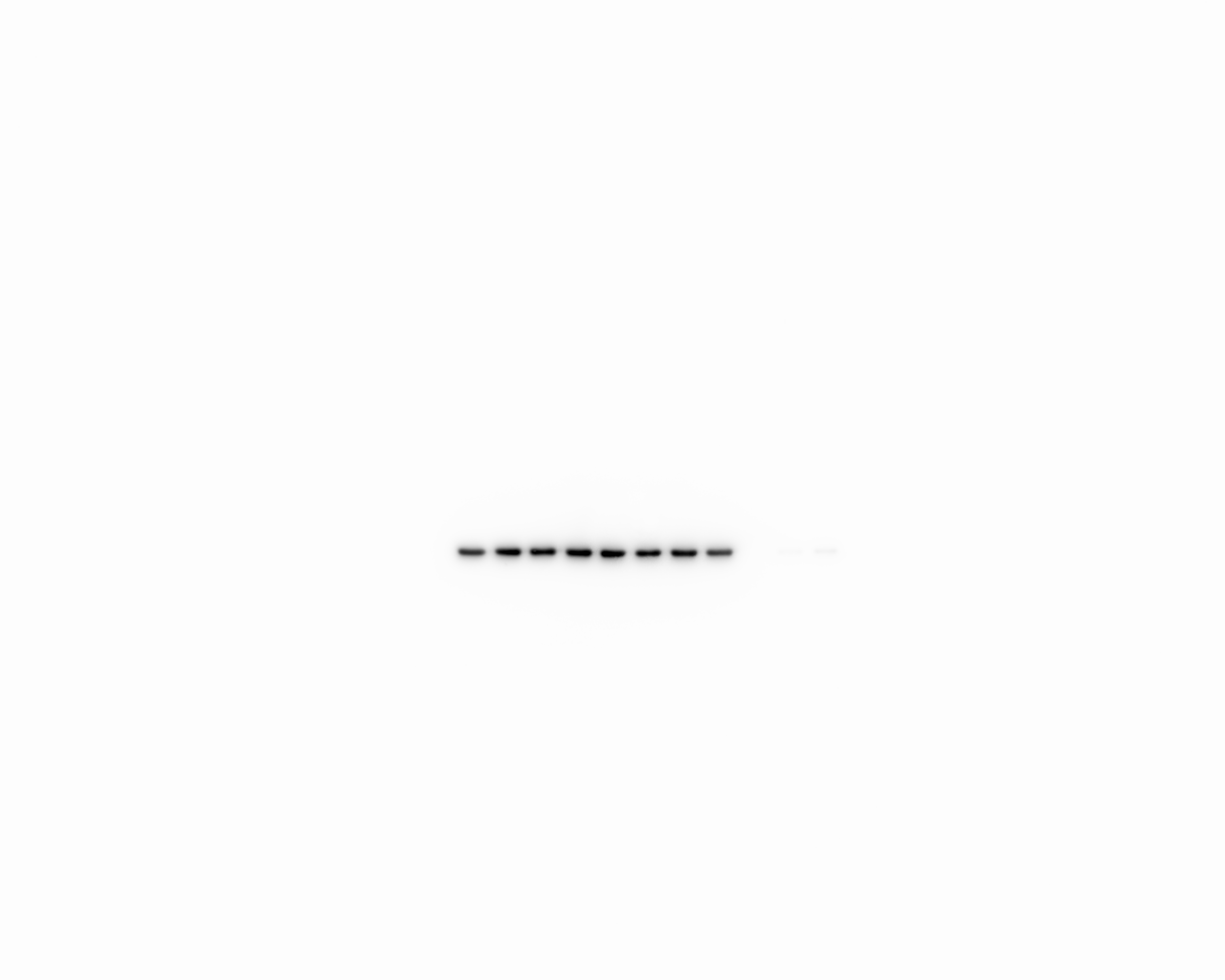

Supplement: Figure 4—source data 2. [file elife-76940-fig4-data2.zip › Figure 4 - source data 2/GAPDH.tif]

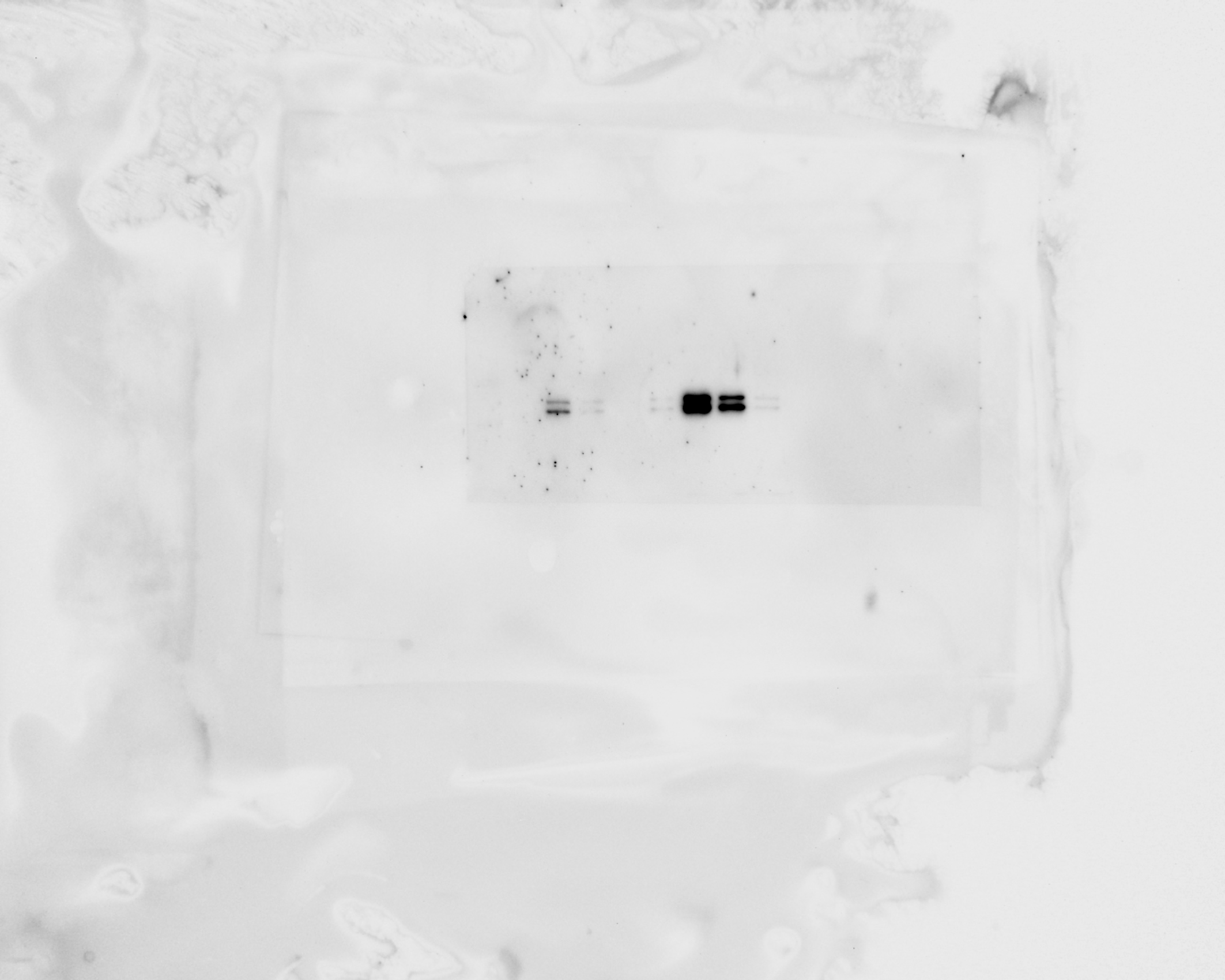

Supplement: Figure 4—source data 2. [file elife-76940-fig4-data2.zip › Figure 4 - source data 2/p-ERK.tif]

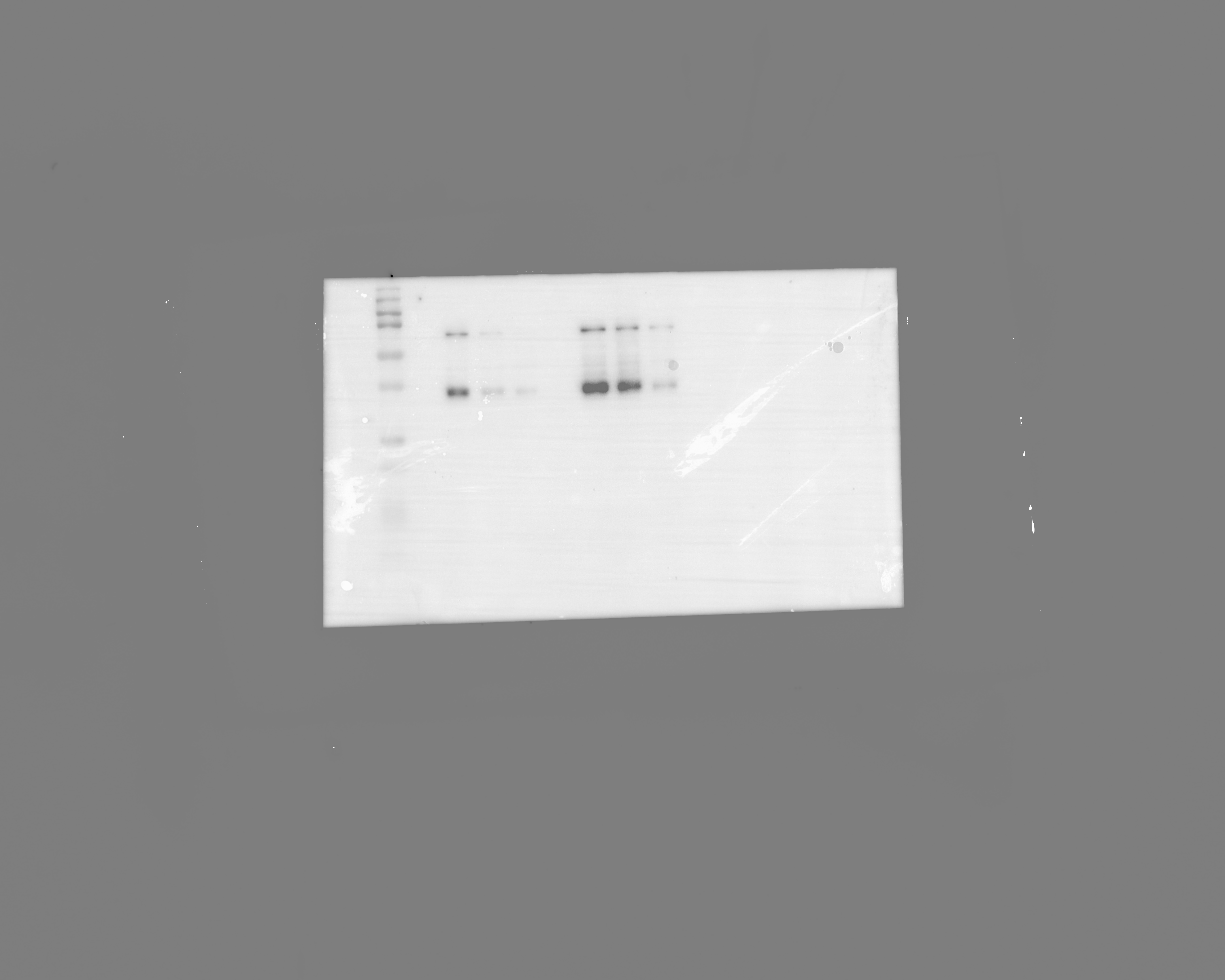

Supplement: Figure 4—source data 2. [file elife-76940-fig4-data2.zip › Figure 4 - source data 2/p-LAT.tif]

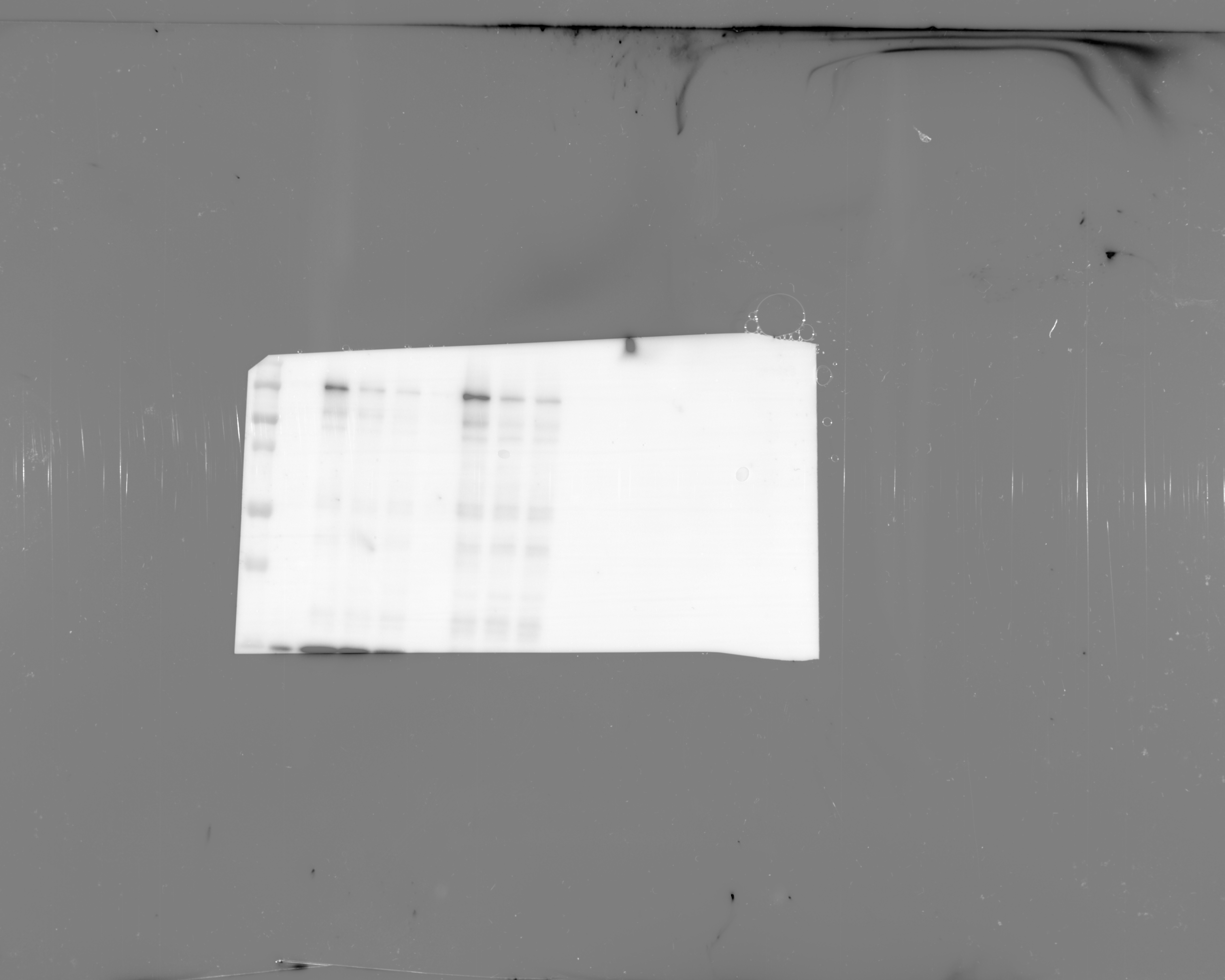

Supplement: Figure 4—source data 2. [file elife-76940-fig4-data2.zip › Figure 4 - source data 2/p-PLC r1.tif]

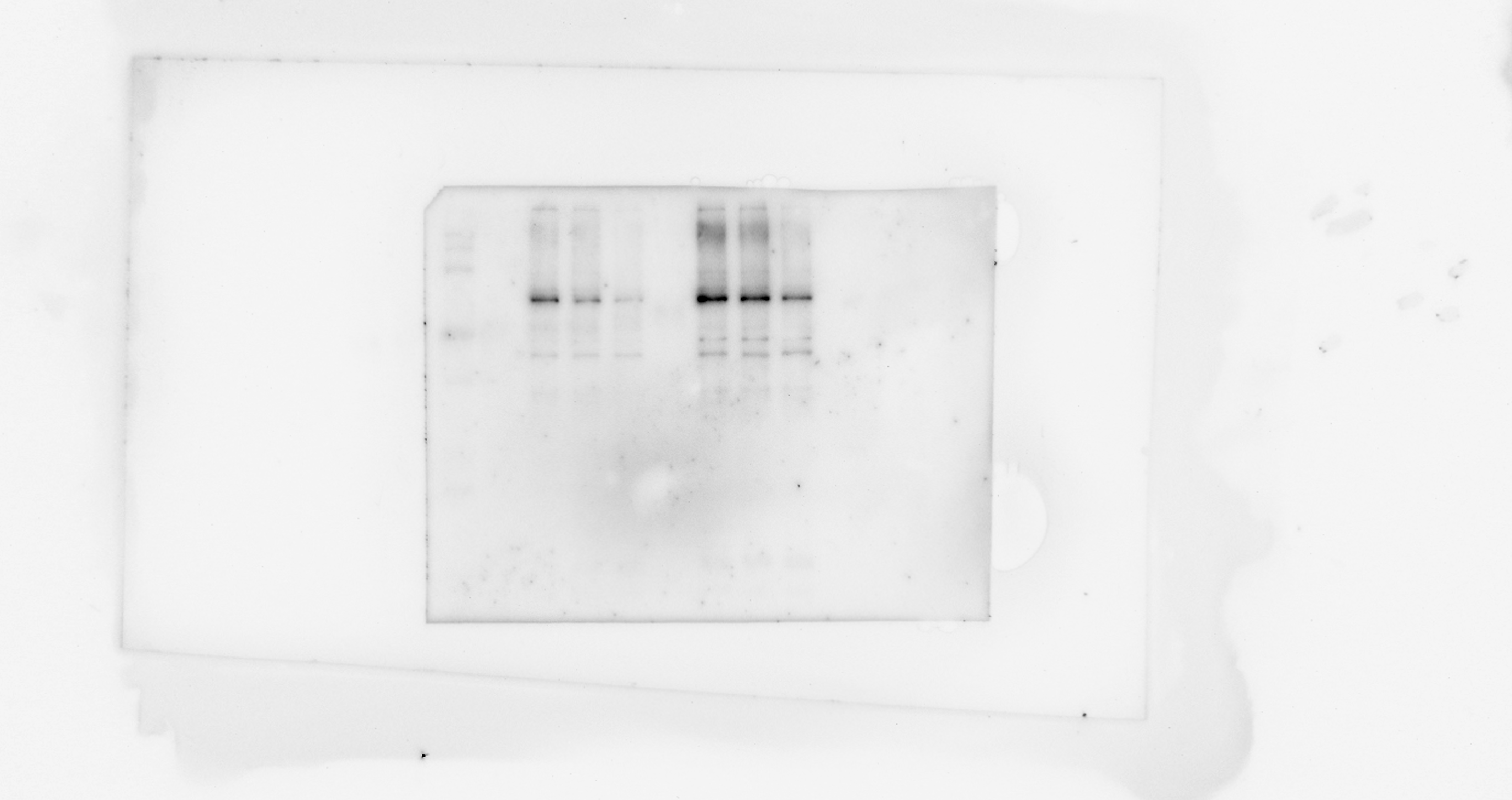

Supplement: Figure 4—source data 2. [file elife-76940-fig4-data2.zip › Figure 4 - source data 2/p-ZAP-70.tif]

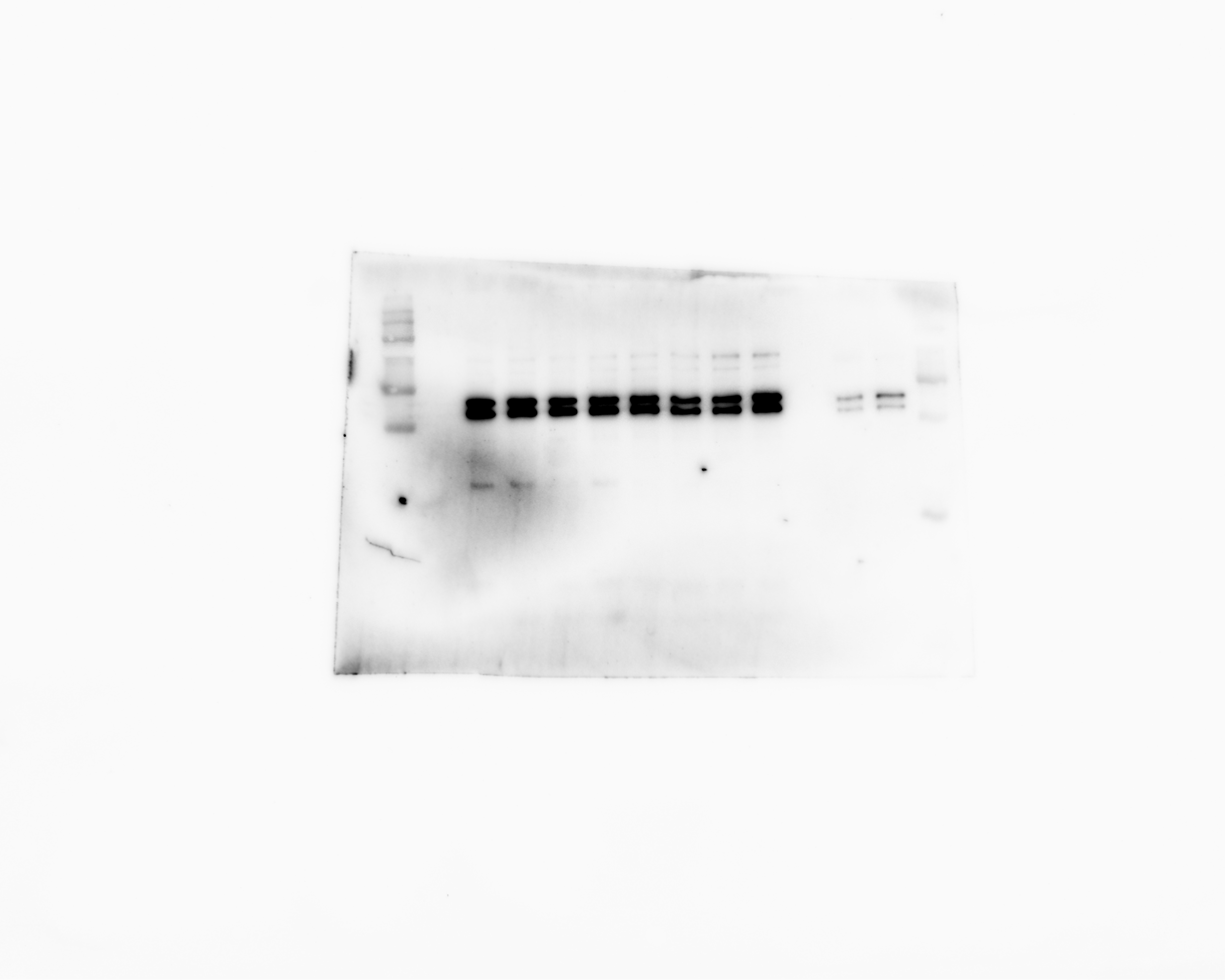

Supplement: Figure 4—source data 2. [file elife-76940-fig4-data2.zip › Figure 4 - source data 2/t-ERK.tif]

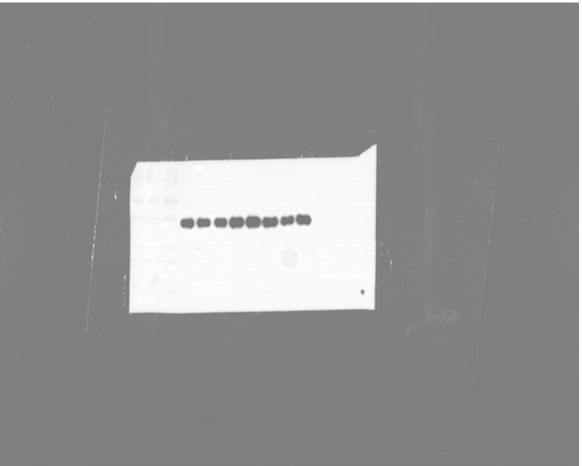

Supplement: Figure 4—source data 2. [file elife-76940-fig4-data2.zip › Figure 4 - source data 2/t-LAT.tif]

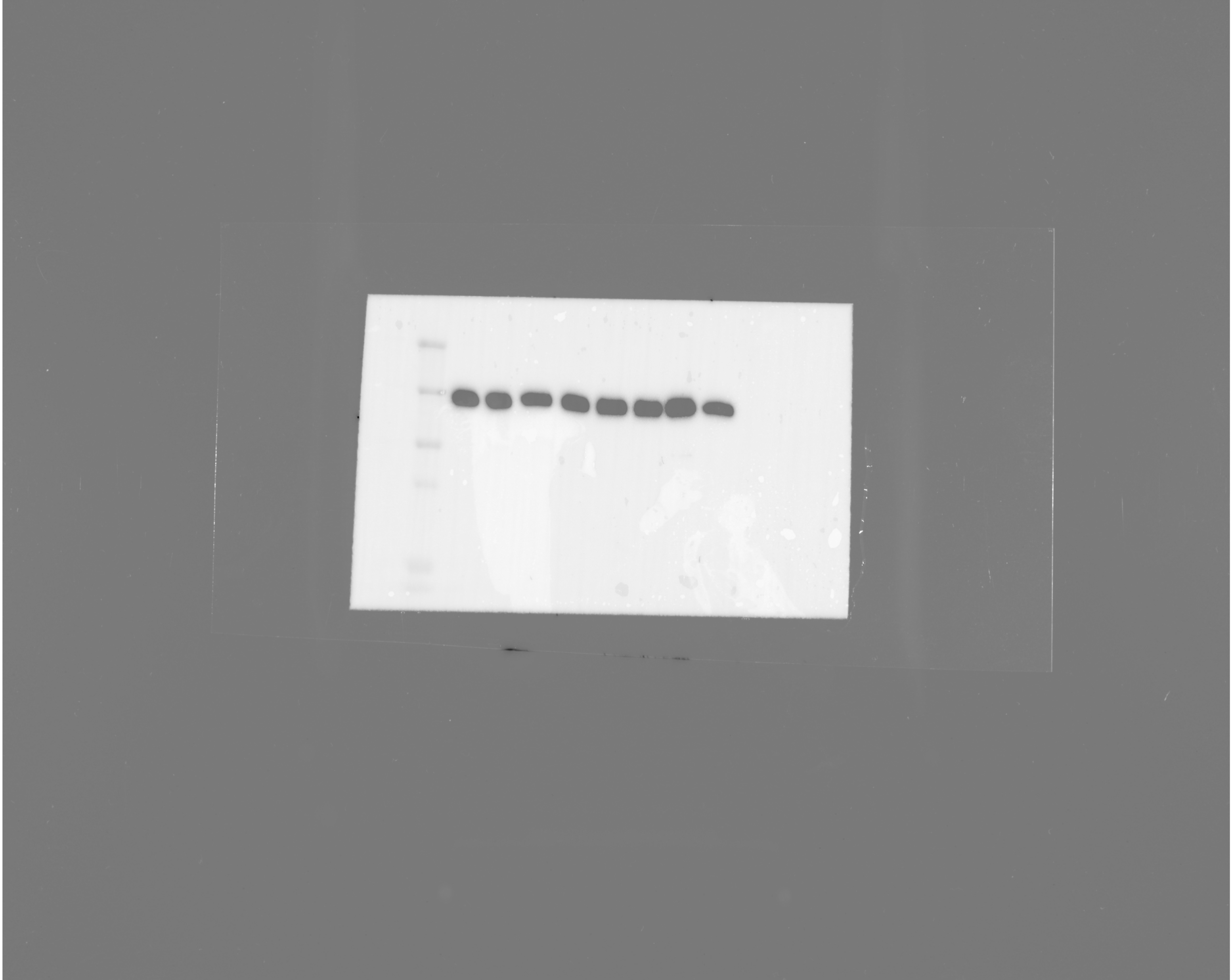

Supplement: Figure 4—source data 2. [file elife-76940-fig4-data2.zip › Figure 4 - source data 2/t-PLC r1.tif]

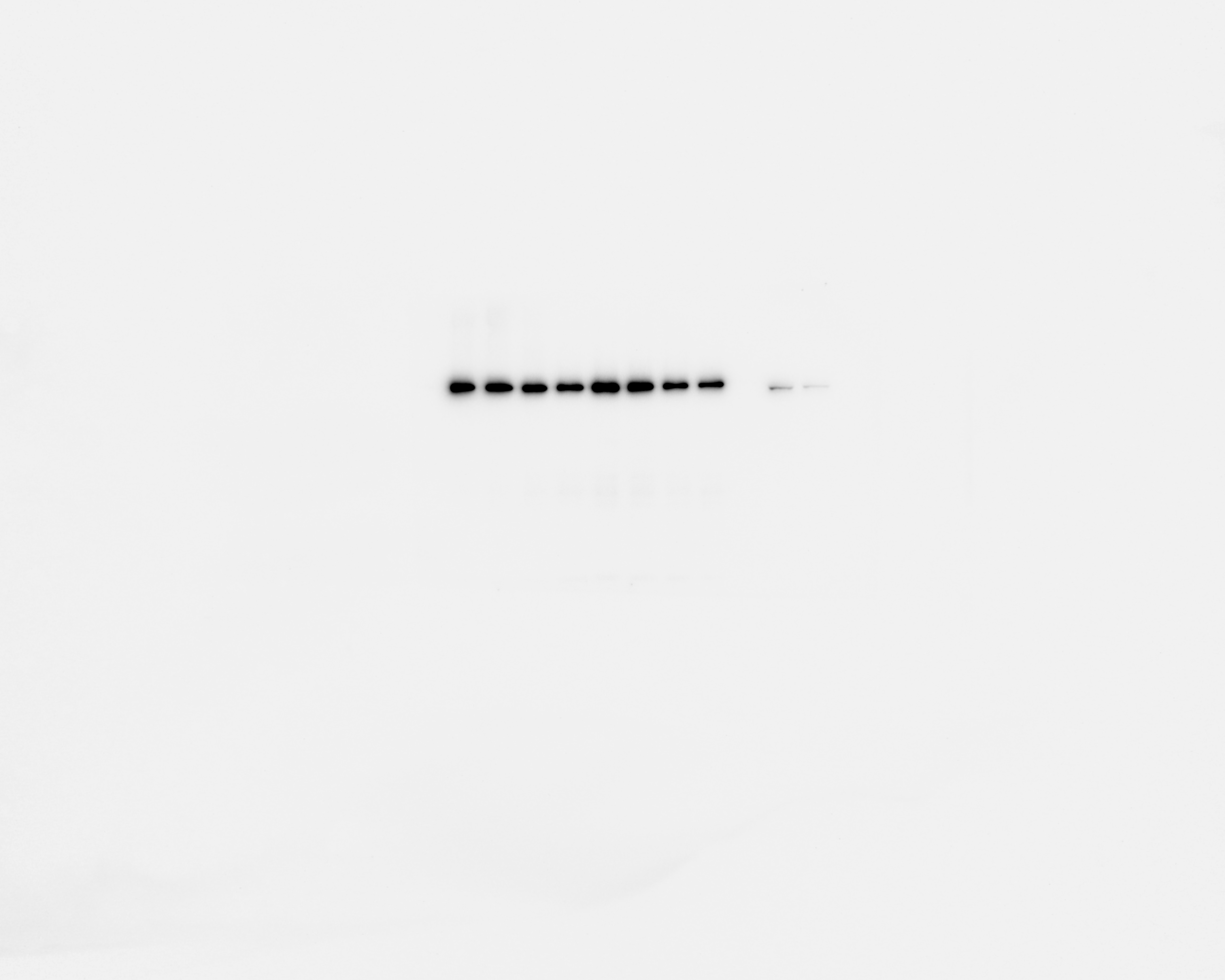

Supplement: Figure 4—source data 2. [file elife-76940-fig4-data2.zip › Figure 4 - source data 2/t-ZAP-70.tif]

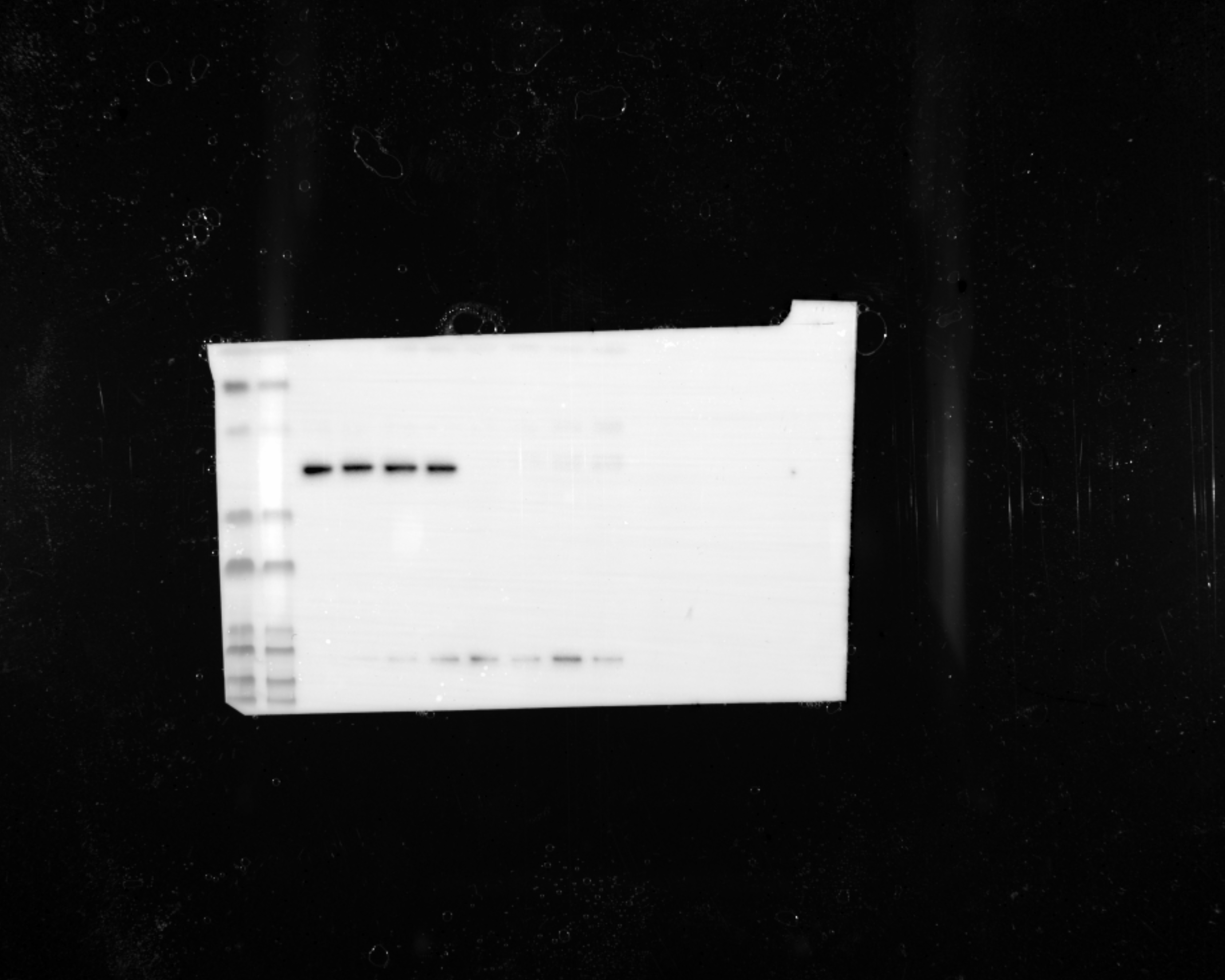

Supplement: Figure 4—source data 3. [file elife-76940-fig4-data3.zip › Figure 4 - source data 3/FAM49B.tif]

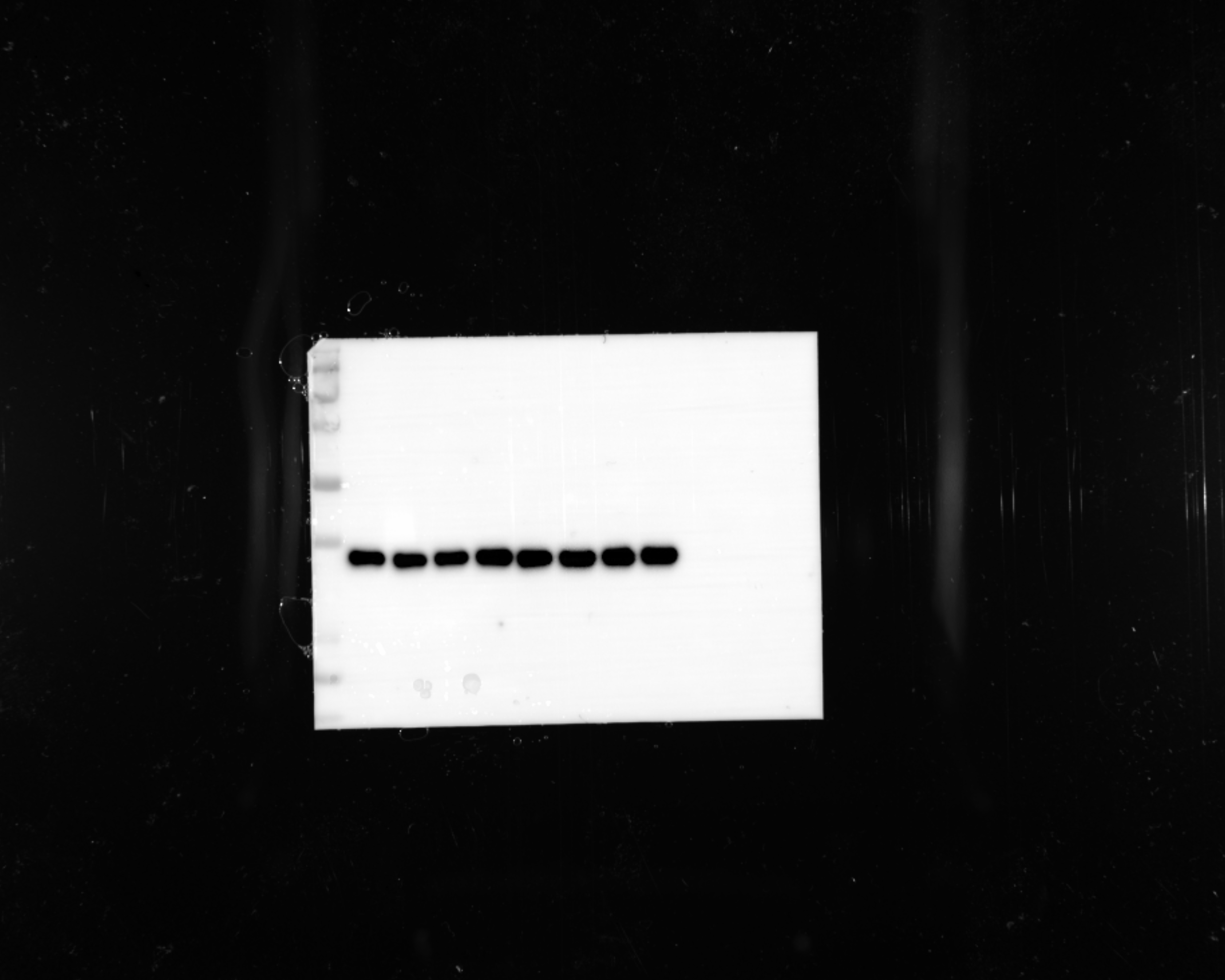

Supplement: Figure 4—source data 3. [file elife-76940-fig4-data3.zip › Figure 4 - source data 3/GAPDH.tif]

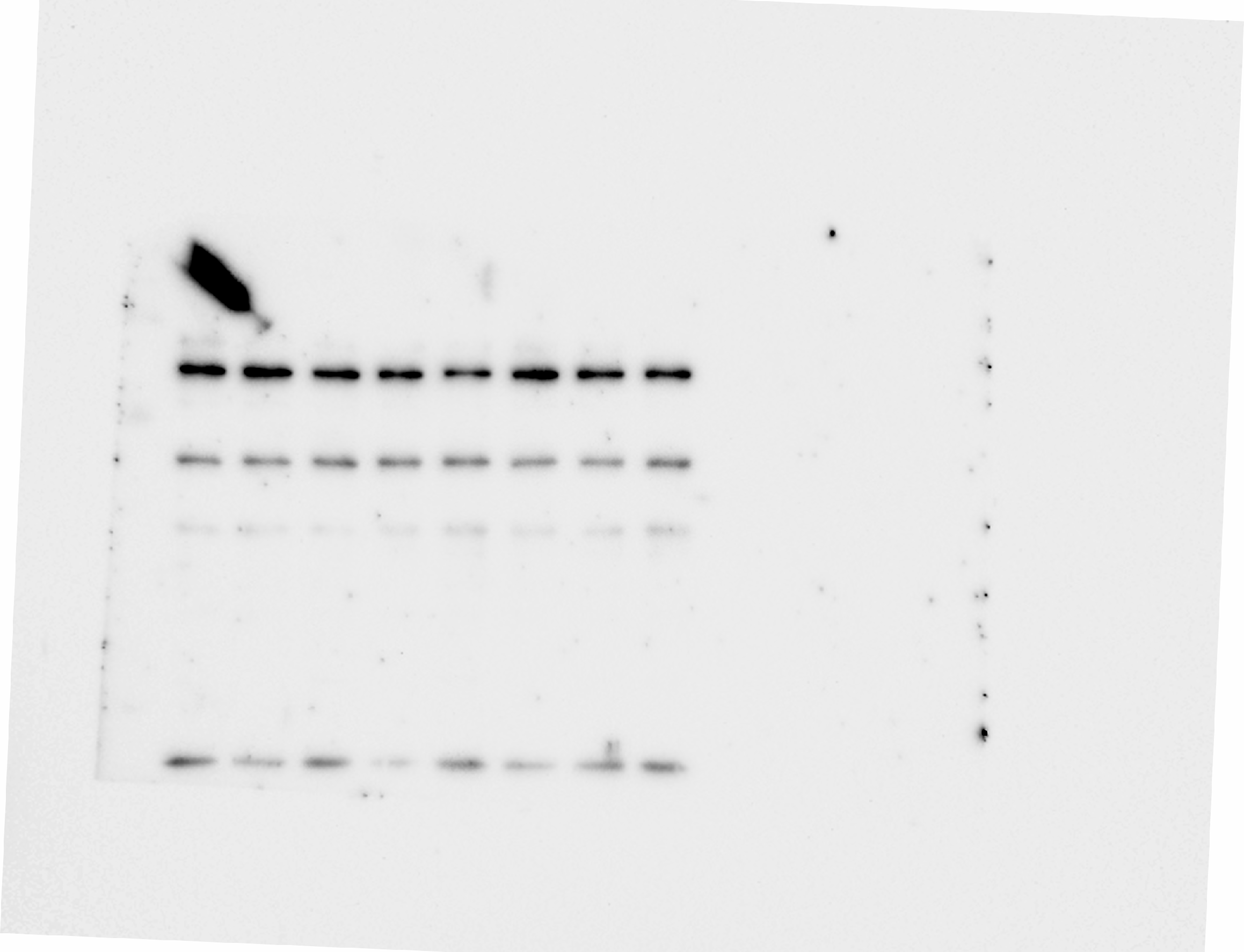

Supplement: Figure 4—source data 3. [file elife-76940-fig4-data3.zip › Figure 4 - source data 3/PAK 123.tif]

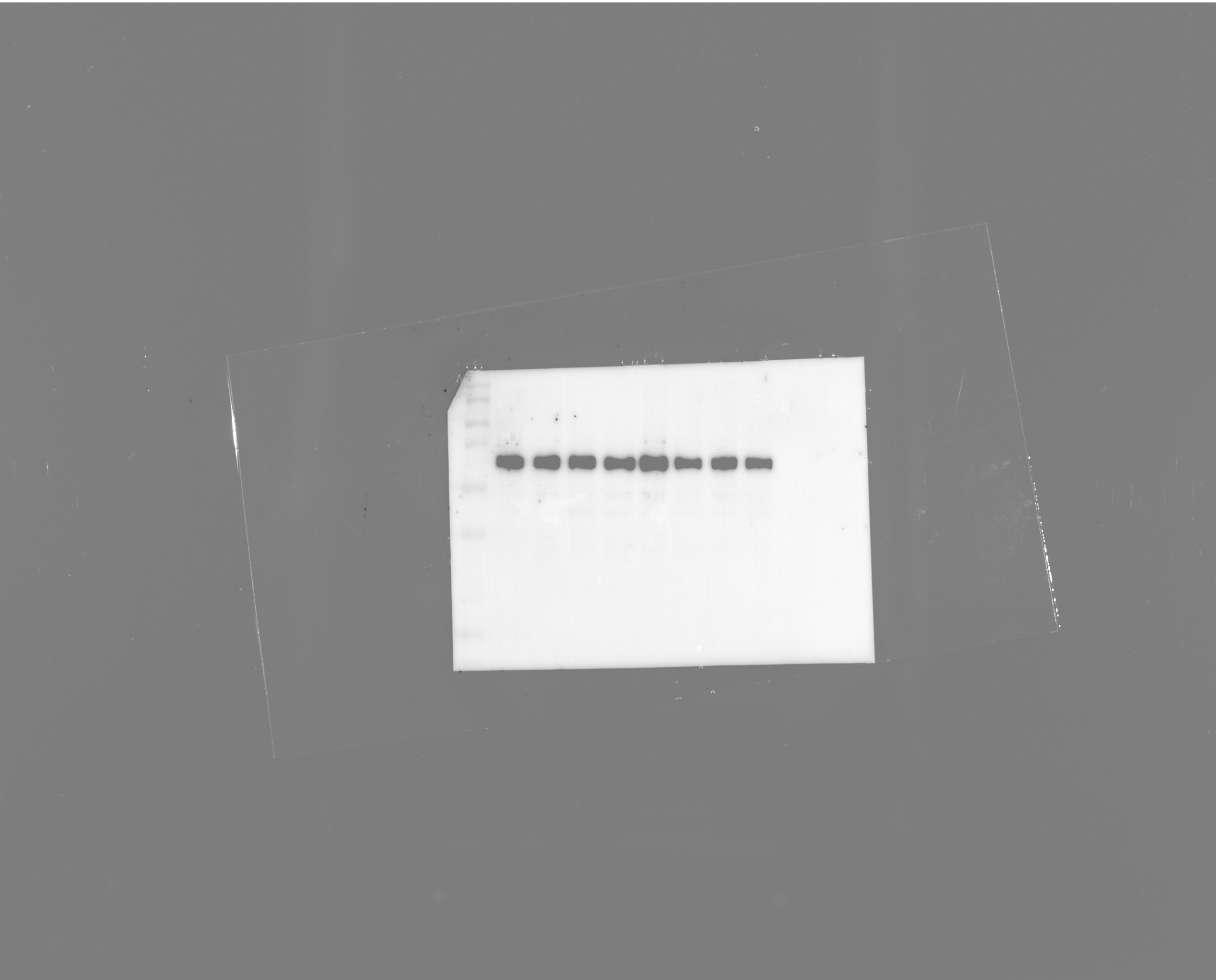

Supplement: Figure 4—source data 3. [file elife-76940-fig4-data3.zip › Figure 4 - source data 3/PAK1.tif]

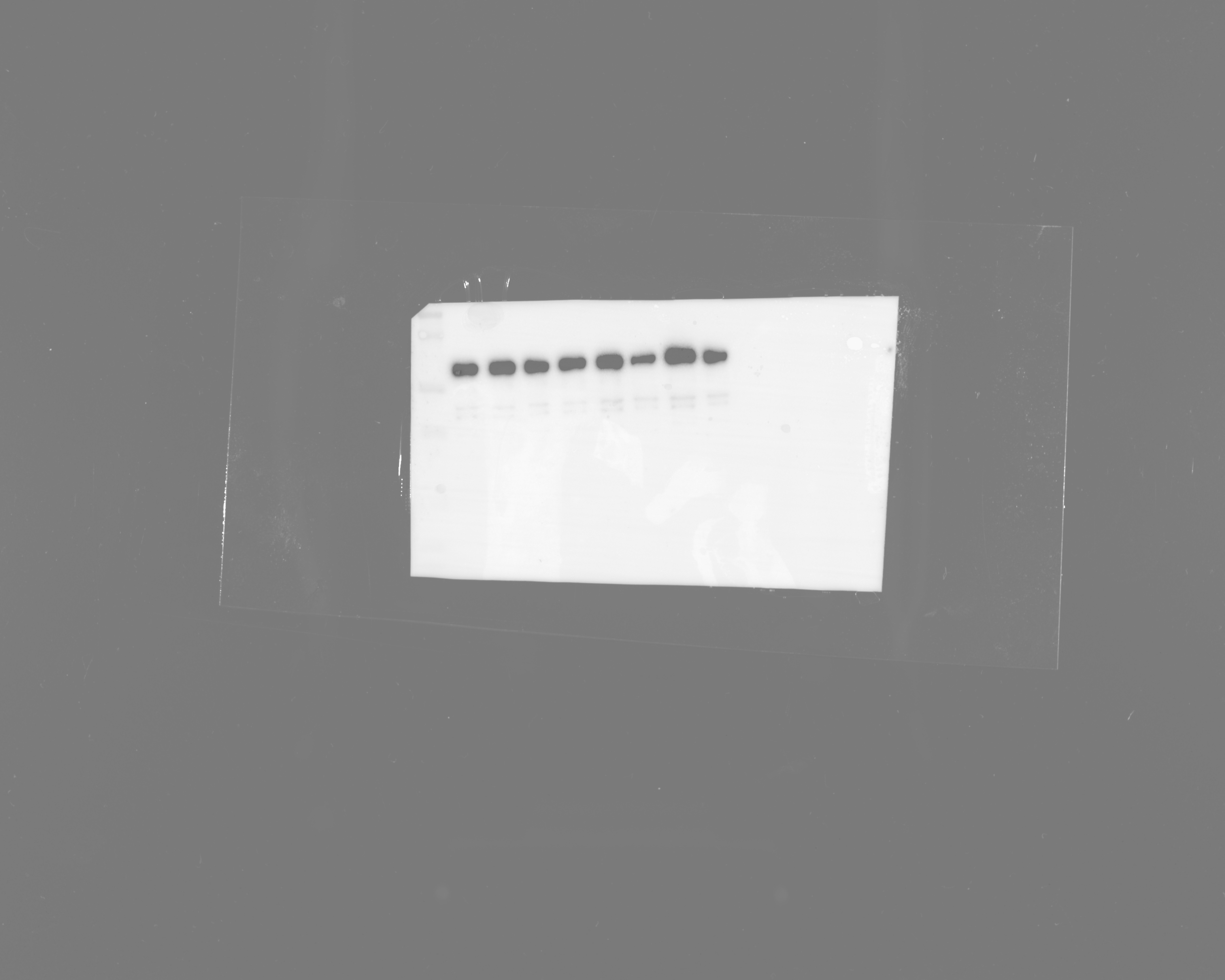

Supplement: Figure 4—source data 3. [file elife-76940-fig4-data3.zip › Figure 4 - source data 3/PAK2.tif]

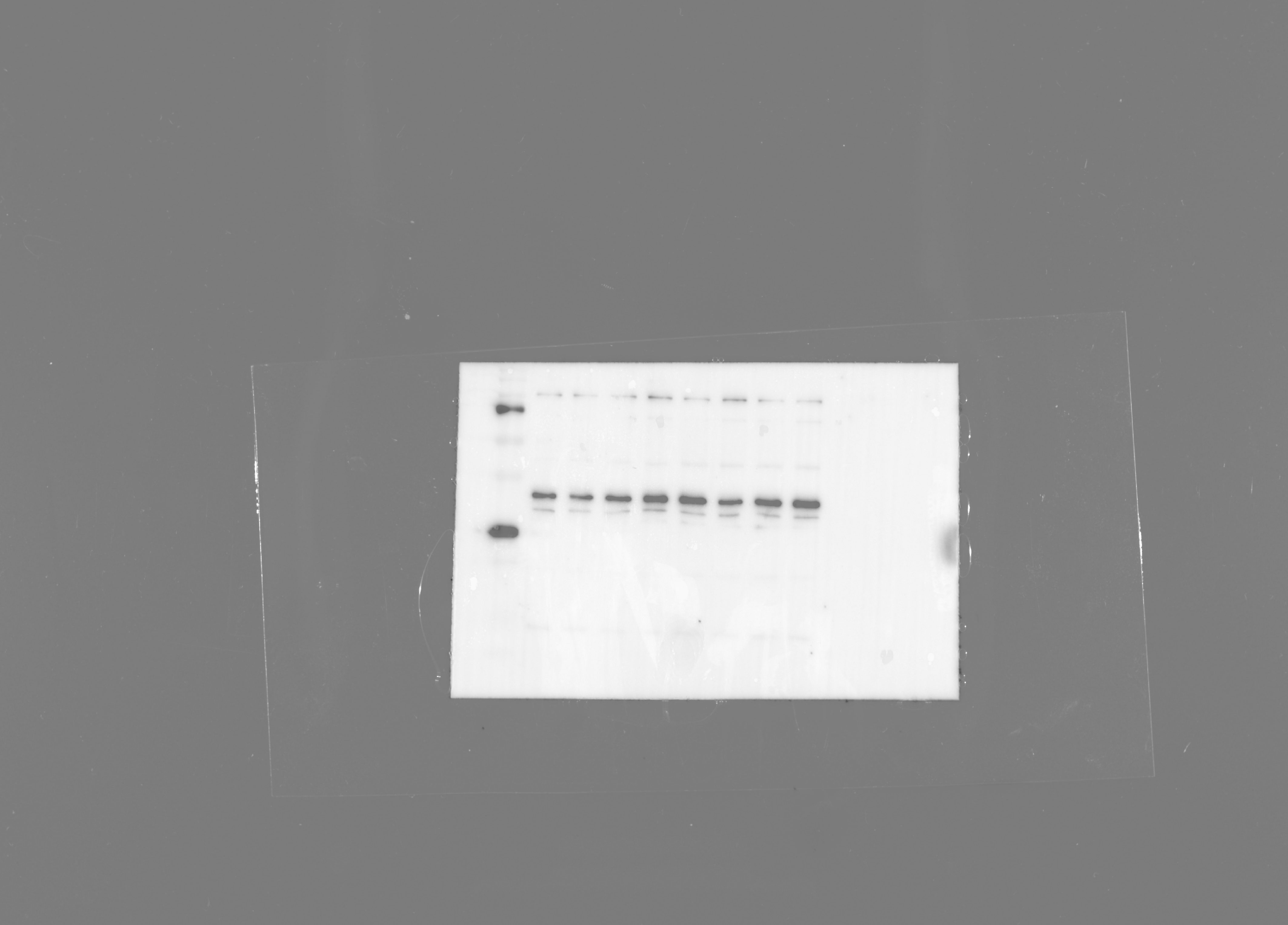

Supplement: Figure 4—source data 3. [file elife-76940-fig4-data3.zip › Figure 4 - source data 3/PAK3.tif]

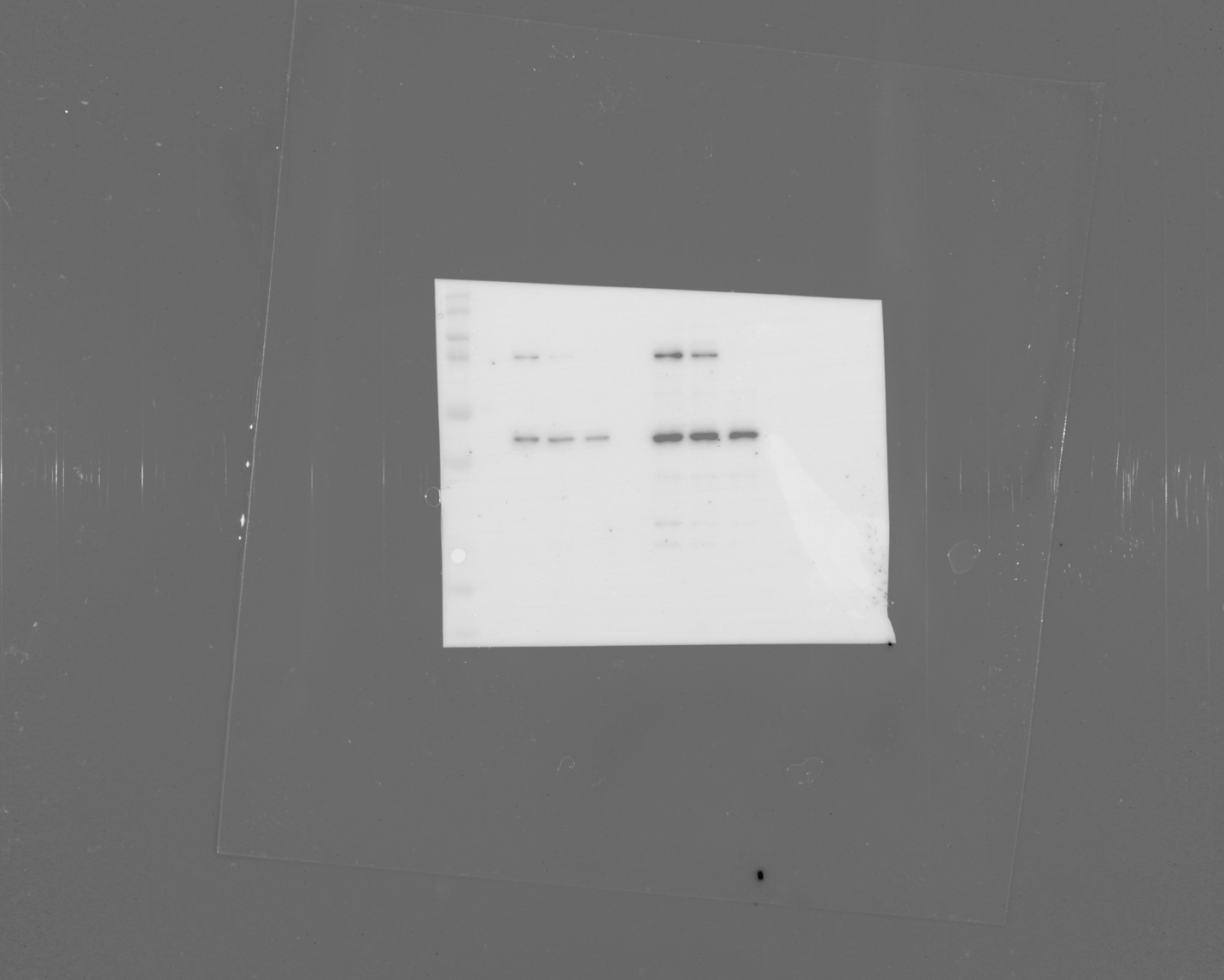

Supplement: Figure 4—source data 3. [file elife-76940-fig4-data3.zip › Figure 4 - source data 3/p-PAK 1(Ser144), 2(Ser 141).tif]

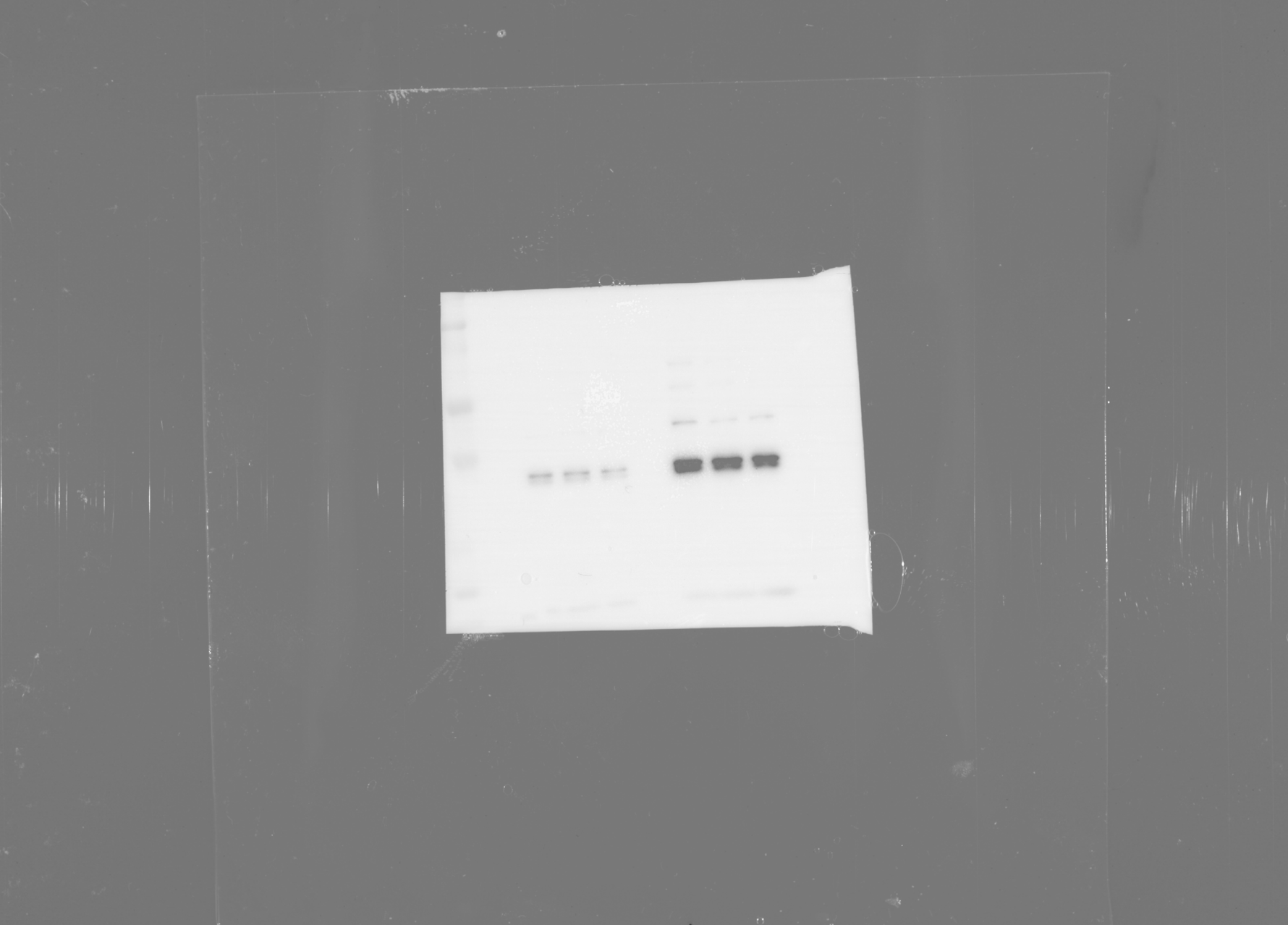

Supplement: Figure 4—source data 3. [file elife-76940-fig4-data3.zip › Figure 4 - source data 3/p-PAK 1(Ser192), 2(Ser 197).tif]

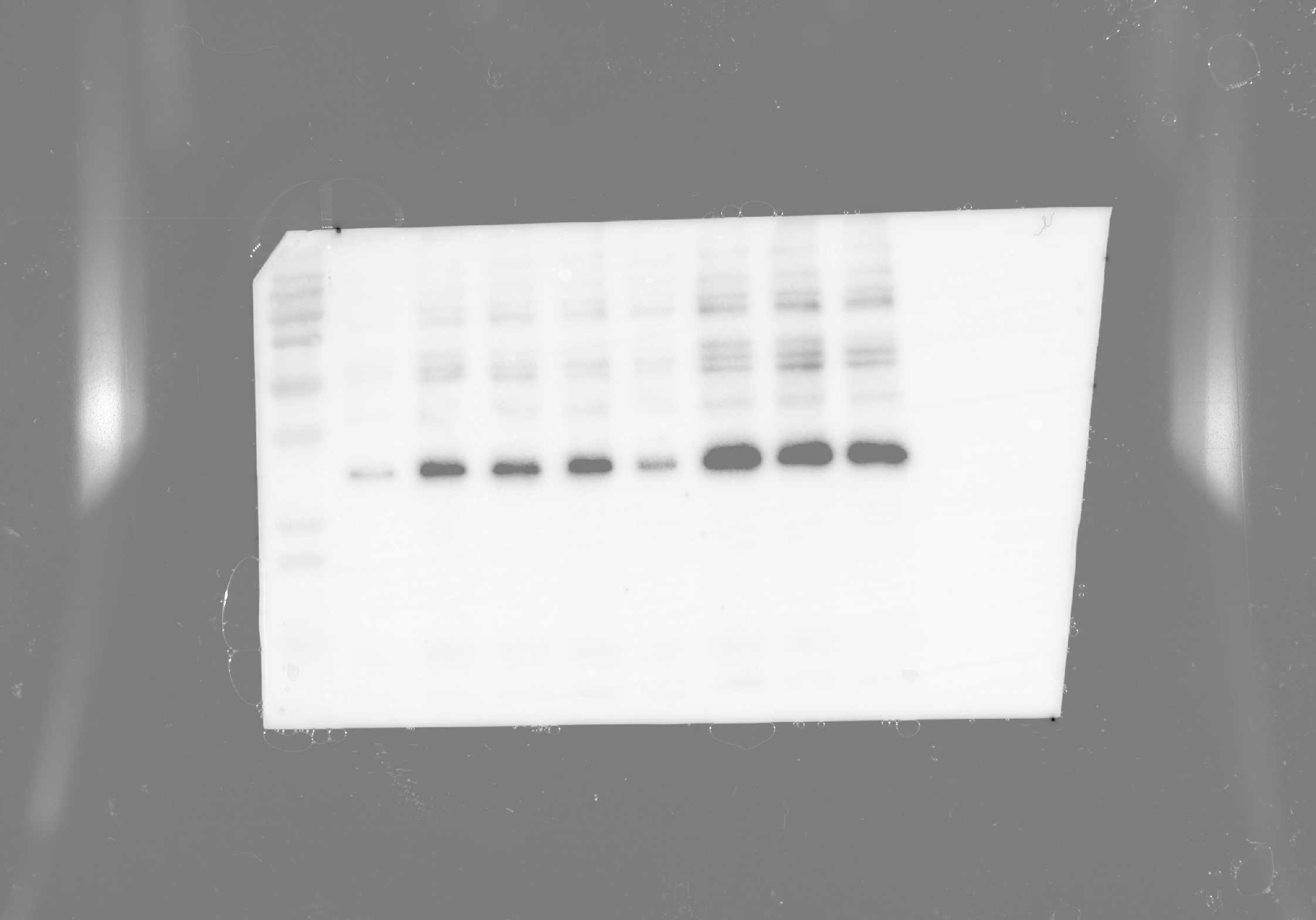

Supplement: Figure 4—source data 3. [file elife-76940-fig4-data3.zip › Figure 4 - source data 3/p-PAK 1(Thr423), 2(Thr402).tif]

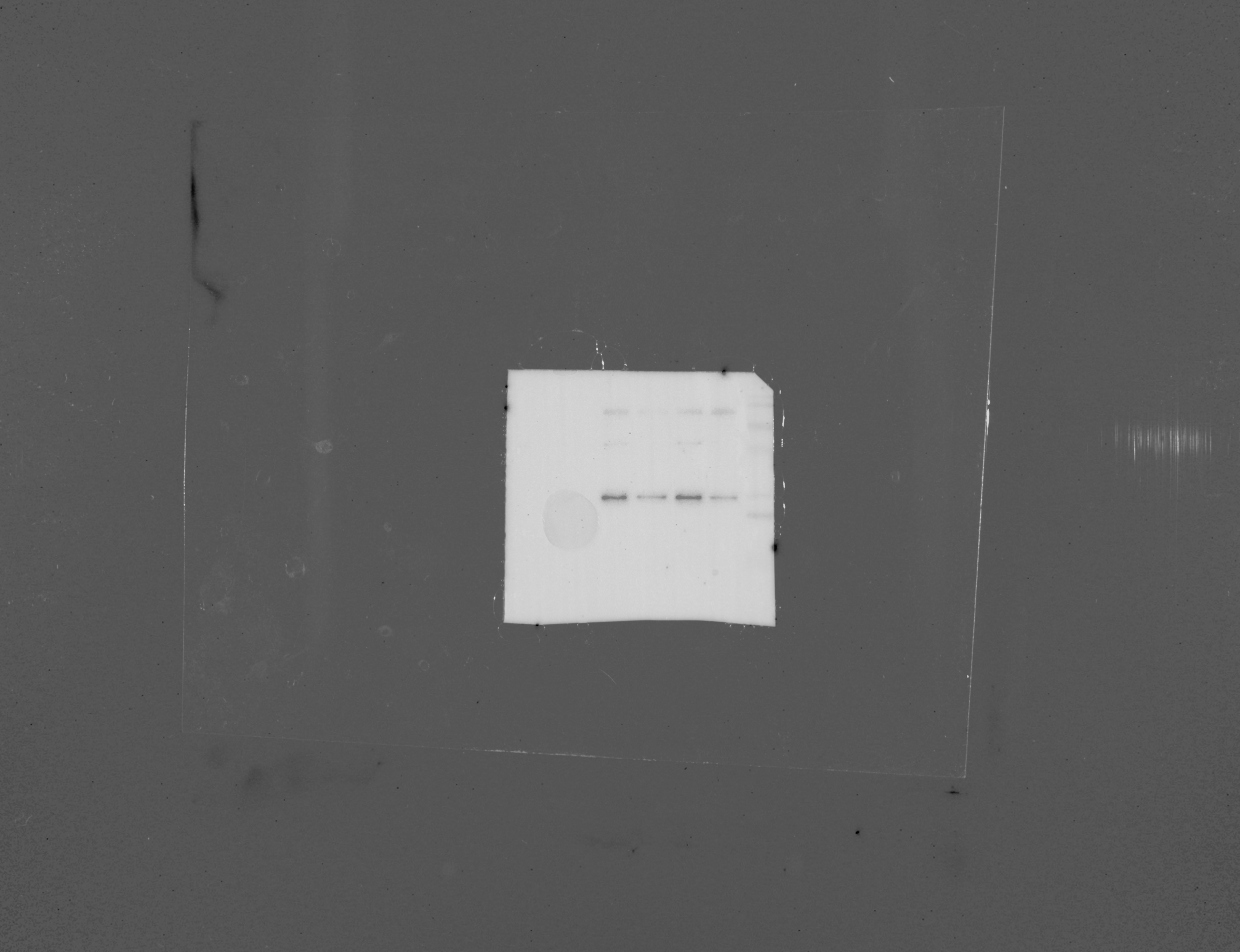

Supplement: Figure 6—figure supplement 2—source data 1. [file elife-76940-fig6-figsupp2-data1.zip › Figure 6-figure supplement 2 - source data 1/Bcl-2.tif]

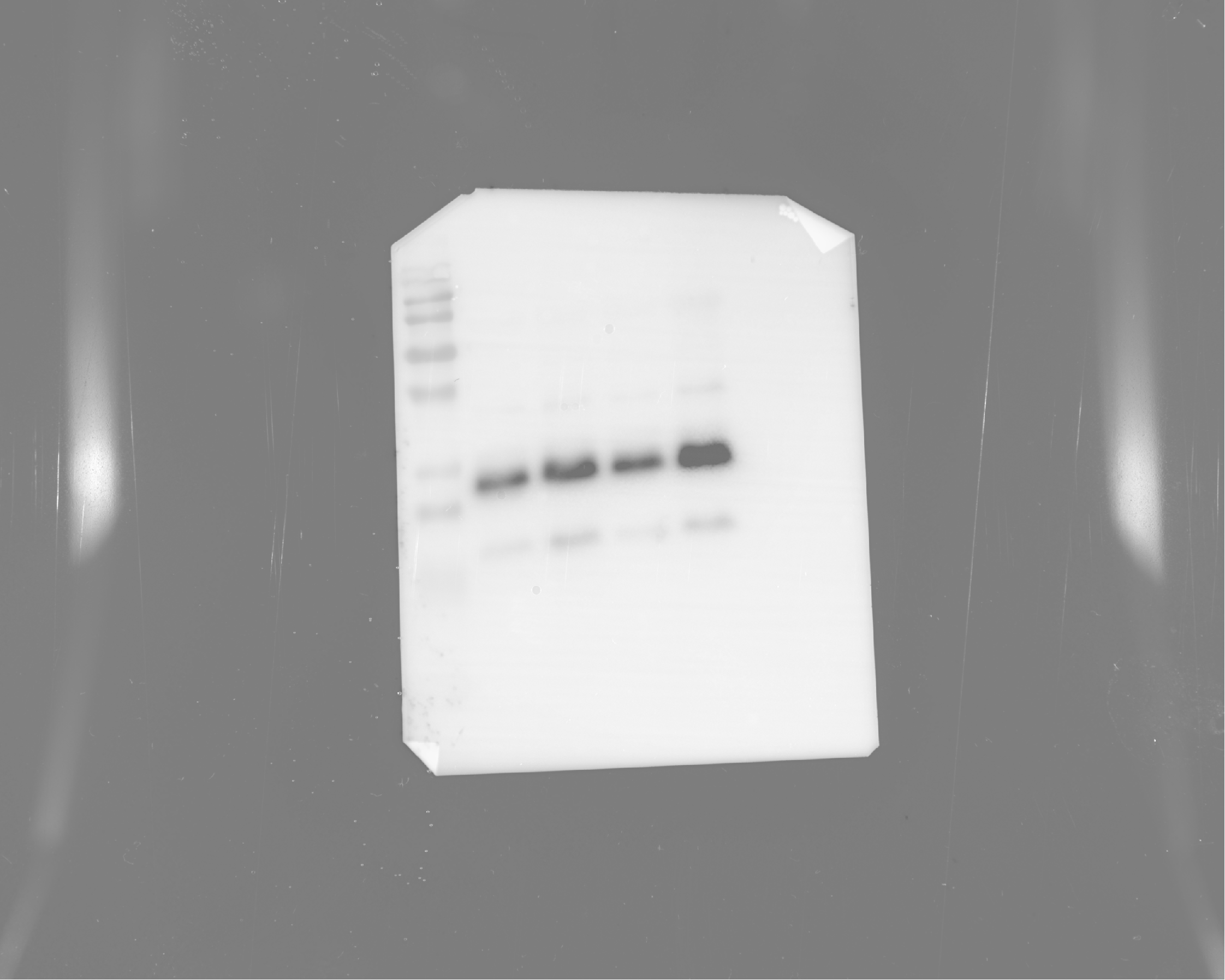

Supplement: Figure 6—figure supplement 2—source data 1. [file elife-76940-fig6-figsupp2-data1.zip › Figure 6-figure supplement 2 - source data 1/BIM.tif]

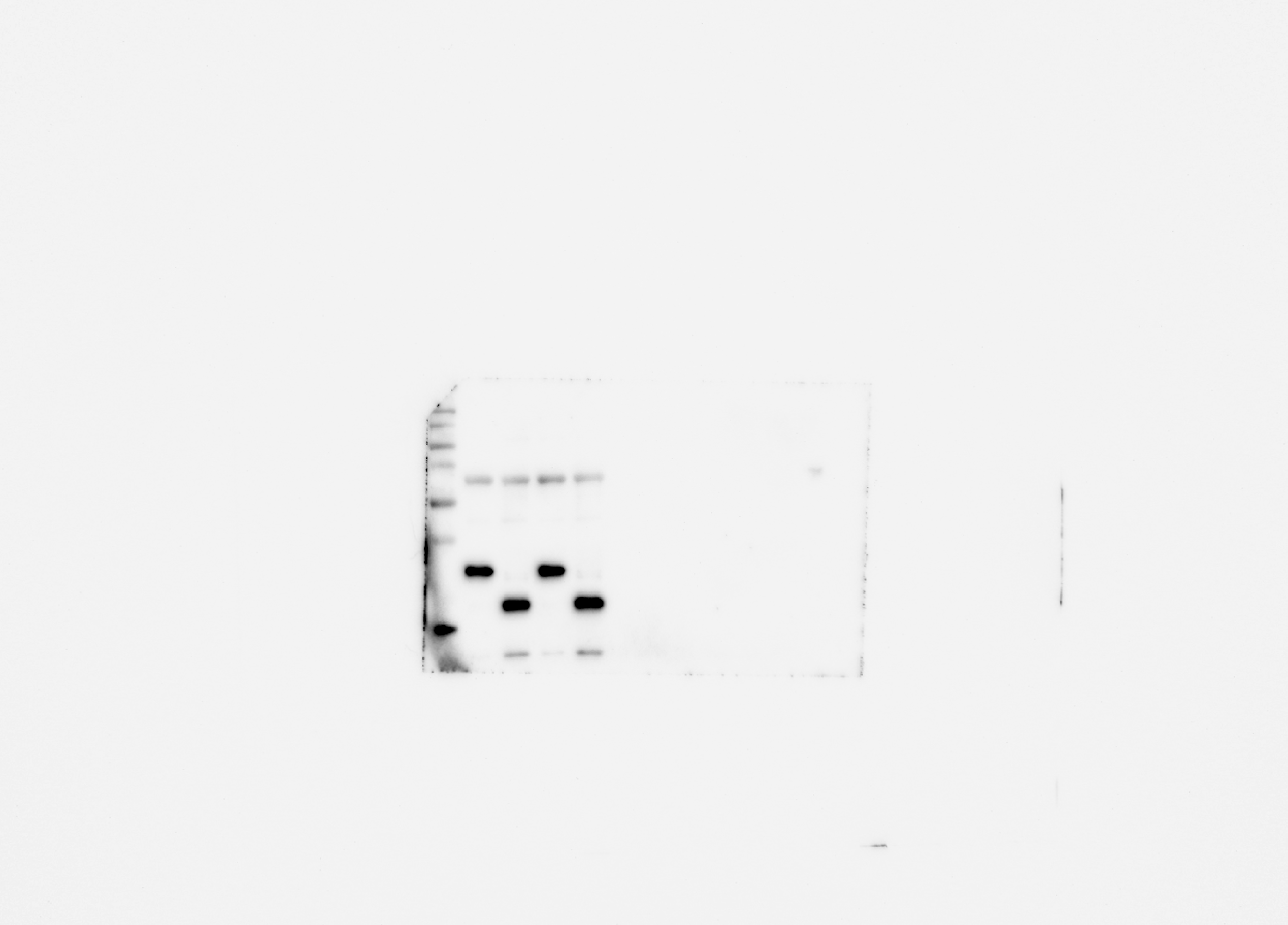

Supplement: Figure 6—figure supplement 2—source data 1. [file elife-76940-fig6-figsupp2-data1.zip › Figure 6-figure supplement 2 - source data 1/FAM49B.tif]

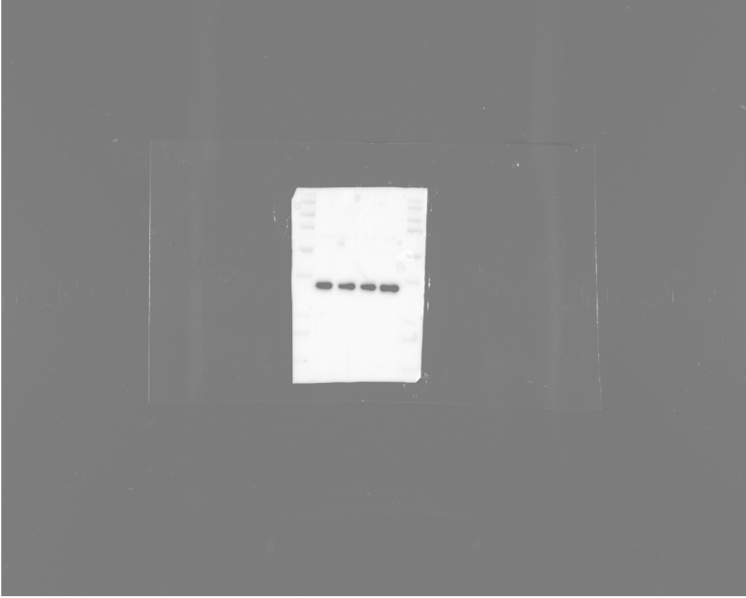

Supplement: Figure 6—figure supplement 2—source data 1. [file elife-76940-fig6-figsupp2-data1.zip › Figure 6-figure supplement 2 - source data 1/GAPDH.tif]
