# Supplementary material for: Fam49b dampens TCR signal strength to regulate survival of positively selected thymocytes and peripheral T cells
Source: eLife. 2024 Aug 19;13:e76940. doi: 10.7554/eLife.76940 (PMC11333044; doi:10.7554/eLife.76940)
Supplement: Figure 4—source data 2. [file elife-76940-fig4-data2.zip › Figure 4 - source data 2/FAM49B.pptx]

## Slide 1
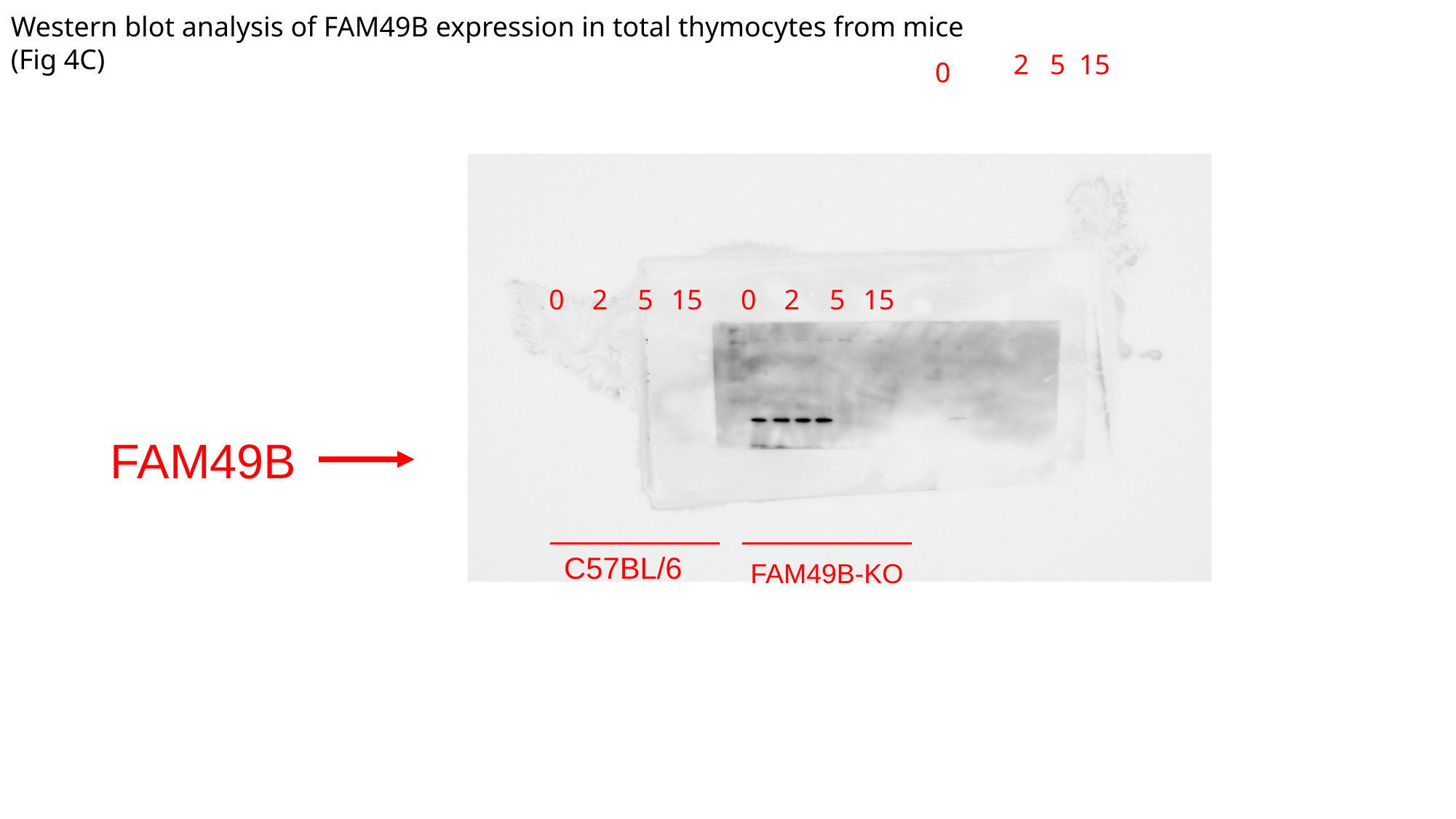

Western blot analysis of FAM49B expression in total thymocytes from mice
(Fig 4C)
2
5
15
0
0
2
5
15
0
2
5
15
FAM49B
C57BL/6
FAM49B-KO
