# Supplementary material for: Fam49b dampens TCR signal strength to regulate survival of positively selected thymocytes and peripheral T cells
Source: eLife. 2024 Aug 19;13:e76940. doi: 10.7554/eLife.76940 (PMC11333044; doi:10.7554/eLife.76940)
Supplement: Figure 4—source data 3. [file elife-76940-fig4-data3.zip › Figure 4 - source data 3/p-PAK 1(Ser192), 2(Ser 197).pptx]

## Slide 1
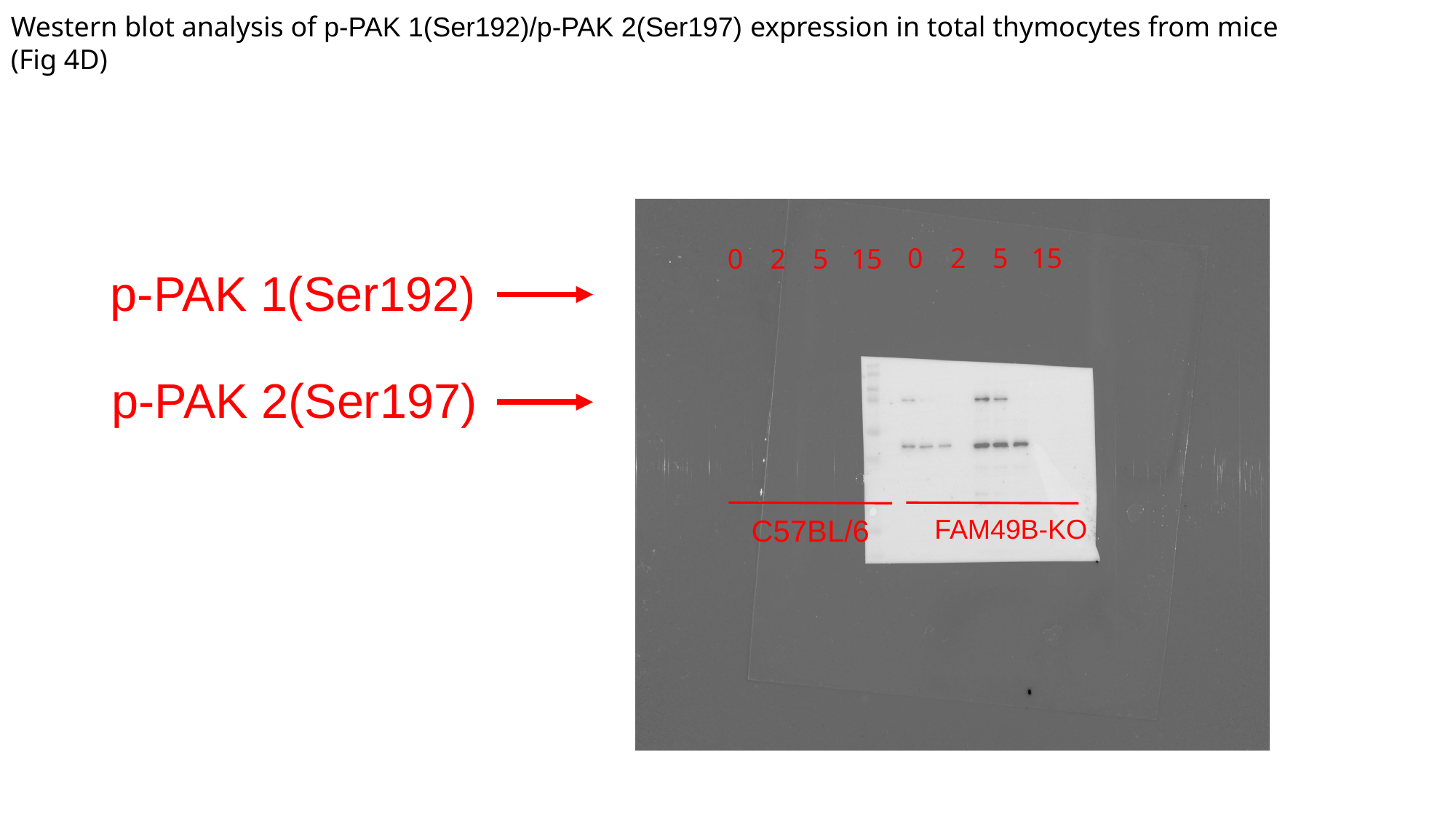

Western blot analysis of p-PAK 1(Ser192)/p-PAK 2(Ser197) expression in total thymocytes from mice
(Fig 4D)
0
2
5
15
0
2
5
15
p-PAK 1(Ser192)
p-PAK 2(Ser197)
FAM49B-KO
C57BL/6
