# Supplementary material for: Fam49b dampens TCR signal strength to regulate survival of positively selected thymocytes and peripheral T cells
Source: eLife. 2024 Aug 19;13:e76940. doi: 10.7554/eLife.76940 (PMC11333044; doi:10.7554/eLife.76940)
Supplement: Figure 6—figure supplement 2—source data 1. [file elife-76940-fig6-figsupp2-data1.zip › Figure 6-figure supplement 2 - source data 1/FAM49B.pptx]

## Slide 1
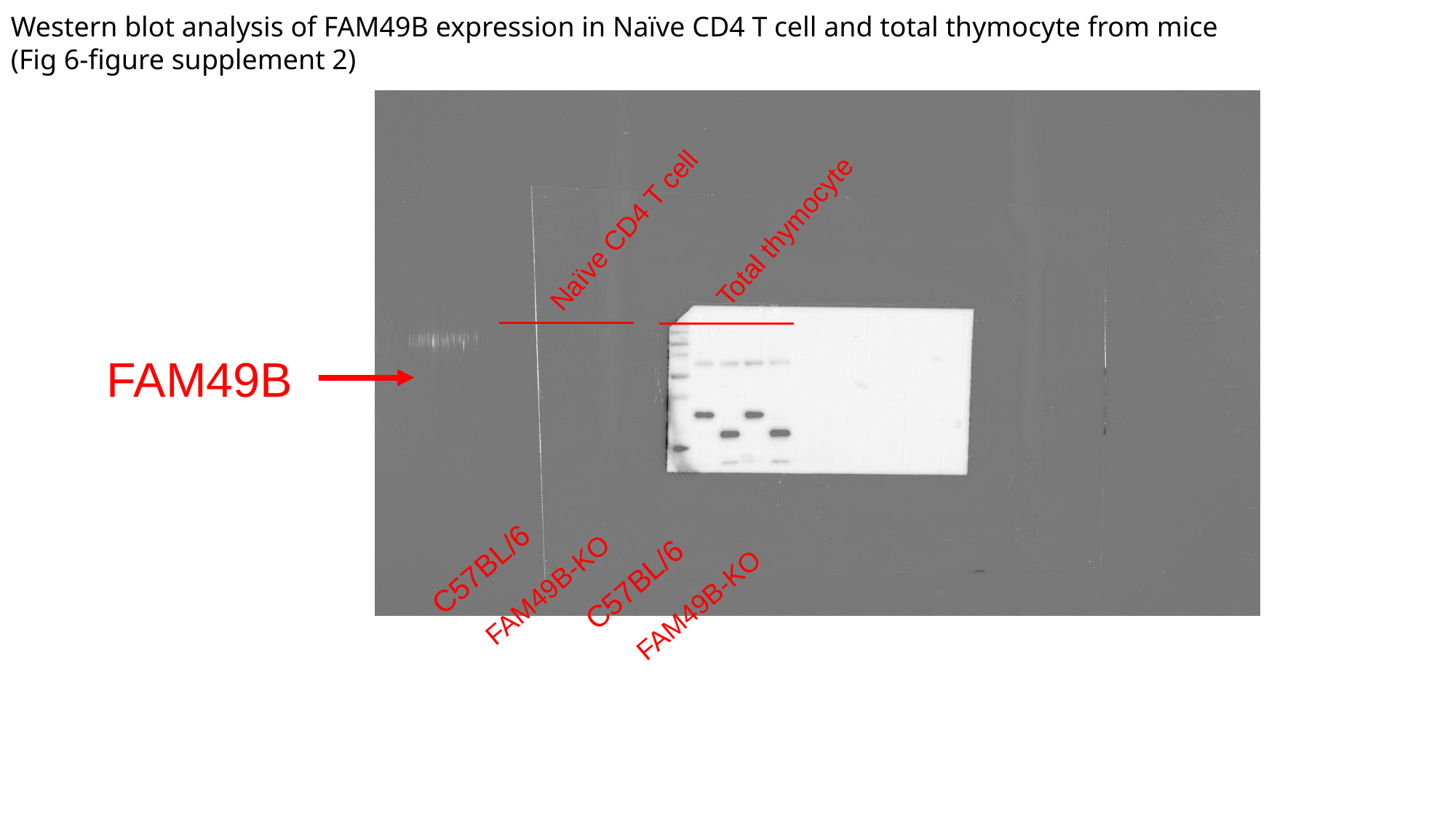

Western blot analysis of FAM49B expression in Naïve CD4 T cell and total thymocyte from mice
(Fig 6-figure supplement 2)
Naïve CD4 T cell
Total thymocyte
FAM49B
C57BL/6
C57BL/6
FAM49B-KO
FAM49B-KO
